# Electrospun Collagen-Coated Nanofiber Membranes Functionalized with Silver Nanoparticles for Advanced Wound Healing Applications

**DOI:** 10.3390/membranes15020039

**Published:** 2025-02-01

**Authors:** Martin Iurilli, Davide Porrelli, Gianluca Turco, Cristina Lagatolla, Alvise Camurri Piloni, Barbara Medagli, Vanessa Nicolin, Giovanni Papa

**Affiliations:** 1 Plastic and Reconstructive Surgery Unit, Cattinara Hospital, Strada di Fiume, 447, 34149 Trieste, Italy; martin.iurilli@phd.units.it (M.I.); gpapa@units.it (G.P.); 2 Clinical Department of Medical, Surgical and Health Sciences, University of Trieste, Strada di Fiume, 447, 34149 Trieste, Italy; 3 Department of Life Sciences, University of Trieste, Via Alexander Fleming 31/B, 34127 Trieste, Italy; 4 Clinical Department of Medical, Surgical and Health Sciences, University of Trieste, Piazza dell’Ospitale 1, 34129 Trieste, Italy; gturco@units.it (G.T.); alvise.camurripiloni@phd.units.it (A.C.P.); bmedagli@units.it (B.M.); nicolin@units.it (V.N.); 5 Department of Life Sciences, University of Trieste, Via Alexander Fleming 22, 34127 Trieste, Italy; clagatolla@units.it

**Keywords:** antibacterial, biocompatibility, collagen, electrospinning, nanofibers, polycaprolactone, silver nanoparticles, wound healing

## Abstract

Complex wounds pose a significant healthcare challenge due to their susceptibility to infections and delayed healing. This study focuses on developing electrospun polycaprolactone (PCL) nanofiber membranes coated with Type I collagen derived from bovine skin and functionalized with silver nanoparticles (AgNPs) to address these issues. The collagen coating enhances biocompatibility, while AgNPs synthesized through chemical reduction with sodium citrate provide broad-spectrum antimicrobial properties. The physical properties of the membranes were characterized using scanning electron microscopy (SEM) and atomic force microscopy (AFM). Results showed the formation of nanofibers without defects and the uniform distribution of AgNPs. A swelling test and contact angle measurements confirmed that the membranes provided an optimal environment for wound healing. In vitro biological assays with murine 3T3 fibroblasts revealed statistically significant (*p* ≤ 0.05) differences in cell viability among the membranes at 24 h (*p* = 0.0002) and 72 h (*p* = 0.022), demonstrating the biocompatibility of collagen-coated membranes and the minimal cytotoxicity of AgNPs. Antibacterial efficacy was evaluated against *Staphylococcus aureus* (SA), *Pseudomonas aeruginosa* (PA), and Vancomycin-resistant *Enterococcus* (VRE), with the significant inhibition of biofilm formation observed for VRE (*p* = 0.006). Overall, this novel combination of collagen-coated electrospun PCL nanofibers with AgNPs offers a promising strategy for advanced wound dressings, providing antimicrobial benefits. Future in vivo studies are warranted to further validate its clinical and regenerative potential.

## 1. Introduction

Wound healing is a multifaceted and dynamic process involving several overlapping stages as follows: hemostasis, inflammation, proliferation, and remodeling. Although the human body possesses the intrinsic ability to repair damaged tissues, various factors, such as infection, underlying health conditions, and compromised immunity, can impede this process, resulting in chronic or hard-to-heal wounds [1]. These include diabetic ulcers, pressure sores, venous leg ulcers, and post-surgical incisions, which pose significant challenges to healthcare systems due to their prolonged healing times, susceptibility to infections, and potential for biofilm formation [2]. Chronic wounds affect millions of people worldwide, contributing to an enormous economic burden on healthcare and significantly diminishing patients’ quality of life [3].

Infection is a critical obstacle in wound healing, as pathogenic bacteria like *Staphylococcus aureus* (SA), *Pseudomonas aeruginosa* (PA), and Vancomycin-resistant *Enterococcus* (VRE) can colonize wounds, leading to persistent inflammation and tissue degradation [4]. Moreover, the formation of bacterial biofilms exacerbates the situation by creating a protective environment that shields the pathogens from antimicrobial agents and the host’s immune system [5,6]. Given these challenges, the development of advanced wound dressings that simultaneously promote tissue regeneration and prevent infection is paramount [7].

Traditional wound dressings, such as gauze, hydrogels, and films, often lack the active functionality needed to prevent and fight local infections while supporting the healing process [8,9]. In response, research has shifted towards multifunctional dressings that not only act as physical barriers but also provide scaffolds that promote cell growth and possess inherent antimicrobial properties [10]. Among these, electrospun nanofiber membranes have attracted substantial interest due to their structural resemblance to the extracellular matrix (ECM) and their potential to incorporate bioactive agents [11].

Electrospinning is a versatile technique that creates nanofibers with a high surface area-to-volume ratio and a porous structure that mimics the ECM [12]. These properties are crucial for facilitating cell adhesion, proliferation, and migration, key processes in tissue regeneration [13]. Polycaprolactone (PCL), a synthetic biodegradable polymer, has emerged as a prominent material in tissue engineering due to its excellent mechanical strength, biocompatibility, and predictable degradation [14]. However, PCL’s hydrophobic nature can limit its effectiveness in promoting cell adhesion and proliferation [15].

To overcome this limitation, natural polymers like collagen, a major component of the ECM, can be integrated into electrospun PCL membranes [16]. Collagen enhances surface hydrophilicity and provides bioactive sites for cell attachment, improving the overall biocompatibility of the fibers [17]. By combining synthetic PCL with collagen, a hybrid scaffold can be created that maintains structural integrity while facilitating the essential cellular activities needed for wound healing [18].

In addition to providing structural support, addressing bacterial infection is critical for effective wound healing. Silver nanoparticles (AgNPs) are widely recognized for their potent antimicrobial properties, which derive from their ability to disrupt bacterial cell membranes, generate reactive oxygen species (ROS), and interfere with microbial DNA replication [19]. Unlike antibiotics, AgNPs can target multiple bacterial structures simultaneously, reducing the risk of bacterial resistance [20]. However, it is essential to carefully control the concentration of AgNPs to avoid cytotoxic effects on human cells, especially during prolonged wound management [21,22].

This study uniquely combines electrospun PCL, bovine-derived Type I collagen, and AgNPs to create a multifunctional nanofiber membrane for advanced wound healing applications. The individual properties of these components are well-documented; PCL provides mechanical support and biodegradability, collagen enhances biocompatibility and cellular activity, and AgNPs offer potent antimicrobial efficacy. However, their integration into a single system represents a novel approach. This combination brings together the structural, bioactive, and antimicrobial features necessary for advanced wound dressings, addressing key challenges in tissue regeneration and infection prevention. To our knowledge, this is the first report on such a composite system, representing a significant step forward in wound care material design.

The objectives of this research are threefold as follows: first, we characterize the physical properties of the nanofiber membranes, including morphology, and surface hydrophilicity; second, we assess their in vitro biological performance in terms of biocompatibility and cytotoxicity using murine 3T3 fibroblasts; and third, we evaluate their antimicrobial efficacy against key wound pathogens, such as SA, PA, and VRE.

The results of this research aim to contribute to the development of advanced wound dressings that address the critical needs of chronic and infected wound management. By comparing collagen-coated nanofiber membranes functionalized with AgNPs, this study offers valuable insights into the design of next-generation materials for wound care in plastic surgery and tissue engineering applications.

## 2. Materials and Methods

### 2.1. Materials

Polycaprolactone (PCL) with a molecular weight of 80,000 Da served as the primary polymer for the electrospinning process [23]. Type I collagen, extracted from bovine skin, was utilized as a coating material to improve the bioactivity and cell affinity of the electrospun nanofiber membranes. Silver nanoparticles (AgNPs) were synthesized via chemical reduction, using silver nitrate (AgNO_3_) as the precursor and sodium citrate as the reducing agent, and were applied as a coating material to improve the antibacterial properties. To dissolve PCL during the electrospinning process, dichloromethane (DCM) and dimethylformamide (DMF) were used as solvents [24]. All products and reagents were of analytical grade, were purchased from Merck (St. Louis, MO USA), and were employed without further purification.

### 2.2. Preparation of Nanofiber Membranes

#### 2.2.1. Electrospinning of PCL-Based Nanofiber Membranes

Electrospinning was employed to fabricate the nanofiber membranes. A 12% (*w/v*) PCL solution was prepared by dissolving 1.2 g of PCL in 10 mL of a DCM and DMF mixture at a 7:3 ratio. The solution was stirred continuously for 24 h at room temperature to ensure complete dissolution [25].

The electrospinning setup consisted of a high-voltage power supply, a syringe pump, and a static flat collector plate. The PCL solution was loaded into a syringe equipped with a 27-gauge needle. To produce uniform nanofibers, the electrospinning parameters were optimized as follows: the applied voltage was set to 17 kV, the distance between the needle and the collector was 28 cm, and the solution flow rate was maintained at 0.6 mL/h. The syringe had an inner diameter of 9.1 mm, and the spinning duration was 2 h.

The electrospun fibers were deposited onto a static flat plate covered with aluminum foil, and after the spinning process was completed, the membranes were left to dry at room temperature for 24 h to allow the complete evaporation of residual solvents.

#### 2.2.2. Collagen Coating

To enhance the biocompatibility of the PCL fibers, the electrospun membranes underwent air plasma low-pressure treatment to improve their hydrophilicity, thus allowing for better collagen adhesion. Plasma treatment was conducted using a PDC-32G plasma cleaner (Harrick Plasma, Ithaca, NY, USA) with a power of 6.8 W for 5 min at 0.13 atm. Following plasma treatment, the membranes were coated by drop-casting 4 µL of a collagen solution (0.1% *w/v*) for 30 min to ensure uniform coating is achieved. The collagen-coated membranes were then left to dry under a fume hood for 24 h to remove excess moisture. The collagen coating aimed to mimic the natural ECM, providing bioactive sites for cell attachment and enhancing the membranes’ overall biocompatibility.

The collagen concentration of 0.1% *w/v* was chosen based on preliminary experiments that demonstrated its ability to enhance biocompatibility while maintaining the structural integrity of the electrospun nanofibers. Although higher concentrations of collagen (e.g., 2–10% *w/v*) are commonly reported in the literature for generating stable nanofibers, these concentrations are typically used in solution preparation for electrospinning rather than coating applications. In contrast, when collagen is used as a coating, as in this study, lower concentrations are generally preferred to facilitate uniform application without compromising surface features. This approach aligns with findings by Deng et al. (2015) [26], who demonstrated that lower collagen concentrations in layer-by-layer (LBL) structured coatings effectively enhance biocompatibility and support wound healing properties (Biomimetic LBL structured nanofibrous matrices assembled by chitosan/collagen to promote wound healing).

#### 2.2.3. Silver Nanoparticle (AgNP) Synthesis and Coating

Silver nanoparticles (AgNPs) were synthesized using the sodium citrate reduction method, also known as Lee-Meisel synthesis [27]. A solution of silver nitrate (AgNO_3_) was prepared by dissolving 0.045 g of AgNO_3_ in 250 mL (180 µg/mL) of deionized water. The solution was then homogeneously stirred until boiling, and 5 mL of sodium citrate tribasic solution (1.1% *w/v*) was added dropwise. The mix was boiling for 1 h under continuous stirring and then cooled to room temperature. The solution turned from transparent to gray, indicating the formation of AgNPs.

The concentration of AgNPs was selected based on literature reports indicating that AgNPs at similar concentrations (25–250 µg/mL), depending on the microorganism [28,29,30,31], effectively inhibit bacterial growth without inducing cytotoxicity. This balance ensures the antimicrobial efficacy of the membranes while maintaining biocompatibility. Our selected concentration reflects an optimal compromise between these two critical factors, allowing the membranes to exhibit significant antibacterial activity alongside minimal reduction in cell viability.

The AgNPs were applied as a coating on the electrospun PCL membranes with the same method followed for collagen coating. A precise volume of 4 µL of the AgNPs aqueous citrate solution (176 ng/µL) was applied to each electrospun PCL membrane disk with a diameter of 5 mm. The membranes were then left to dry at room temperature for 24 h. Then, the membranes were rinsed with deionized water to remove any unbound nanoparticles. No additional solvents were employed during the process.

To improve the uniformity of the coating, methods such as the centrifugation of the AgNP solution prior to deposition are being explored. These efforts aim to reduce the potential clustering of AgNPs and achieve a more homogeneous dispersion across the membrane surface.

### 2.3. Characterization of Nanofiber Membranes

#### 2.3.1. Morphological Analysis

The morphology of the electrospun nanofiber membranes was examined using scanning electron microscopy (SEM). The membranes were placed on aluminum stubs coated with double side carbon tape and were sputter-coated with gold (using the Emitech K550X sputter coater, Quorum Technologies, Lewes, UK) to achieve electrical conductivity before imaging. SEM was performed using the Quanta250 Scanning Electron Microscope (FEI, Hillsboro, OR, USA) with an acceleration voltage of 15 kV, and images were captured at various magnifications to assess the fiber diameter, surface topography, and distribution of collagen and silver nanoparticles. The average fiber diameter was measured using Gwyddion^®^ (version 2.67) and ImageJ^®^ (version 1.54d) software by analyzing multiple SEM images for each sample type.

The uniformity of the collagen coating was analyzed using confocal microscopy. Bovine Type I collagen, labeled with fluorescein isothiocyanate (FITC), was applied, allowing the direct visualization of the coating on the nanofiber membranes. SEM in a backscattered electron mode was employed to evaluate the dispersion of AgNPs on the membrane surface. This technique, leveraging the high atomic number contrast of silver, enabled the differentiation between the AgNPs and the polymeric matrix, providing insights into their spatial distribution.

The AgNPs used in this study, as previously mentioned, were synthesized using the well-established Lee-Meisel method [27], which employs citrate as a reducing agent. This technique is widely regarded as a standardized and reproducible approach for generating stable and monodisperse nanoparticles. While TEM imaging was not performed due to limitations in equipment availability, the Lee-Meisel method has been extensively validated in the literature. Studies such as those by Deshpande et al. (2021) [32] and Wan et al. (2013) [33] confirm that AgNPs synthesized under similar conditions typically exhibit a spherical morphology with controlled size distribution. The absence of TEM images is acknowledged as a limitation; however, the standardized synthesis and characterization protocols employed in this study ensure the consistency and reliability of the AgNPs used. Future studies will include direct TEM imaging to further ensure the verification of the nanoparticle morphology and size.

Atomic force microscopy (AFM) was used to evaluate the surface roughness of the collagen-coated fibers. AFM (Solver Pro-M by NT-MDT with probe model SCM-PIT-V2) images were collected in tapping mode, and surface roughness parameters, such as the arithmetic mean roughness (Ra) and root mean square roughness (Rq), were calculated to quantify the impact of the collagen and AgNP coating on the fibers’ topography.

#### 2.3.2. Hydrophilicity and Water Absorption

The surface hydrophilicity of the membranes was evaluated using contact angle measurements. A 4 µL droplet of deionized water was placed on the membrane surface, and the contact angle was measured on images acquired with an optical microscope (Leica MZ16, Microscope Central, Feasterville, PA, USA) equipped with a 45° titled mirror and a digital camera (Leica DFC 320, Microscope Central, Feasterville, PA, USA), through which it was possible to display the profile of the liquid drop on the membrane sample, using ImageJ^®^ software.

Water absorption tests were conducted by immersing the membranes in deionized water at 37 °C for 24 h. The membranes were weighed before and after immersion, and the swelling ratio was calculated using the following formula:

Swelling (%) = ((W_s_ − W_d_)/W_d_) × 100

where W_s_ is the weight of swollen membranes and W_d_ is the weight of dry membranes. This assessment provided insights into the membranes’ ability to absorb wound exudate, as maintaining a moist environment is, in fact, optimal for wound healing.

### 2.4. In Vitro Biological Evaluation

The biocompatibility of the membranes was assessed using 3T3 murine fibroblasts. The fibroblasts were cultured in high-glucose Dulbecco’s Modified Eagle Medium (DMEM) supplemented with FBS 10%, l-glutamine 2 mM, penicillin 100 U/mL, and streptomycin 0.1 mg/mL at 5% pCO2 and at 37 °C. For the biocompatibility assay, the membranes were sterilized under UV light for 30 min on each side and placed on 24-well plates. Approximately 5 × 10^4^ fibroblasts were seeded onto each membrane and incubated at 37 °C with 5% CO_2_ for 24, 48, and 72 h.

Cell viability was evaluated using the MTT assay. After the incubation period, the membranes were rinsed with PBS, and a 20 µL of MTT solution (5 mg/mL) was added to each well. The plates were incubated for 4 h at 37 °C, followed by the addition of dimethyl sulfoxide (DMSO) to dissolve the formazan crystals. The absorbance was measured at 570 nm using a microplate reader, with higher absorbance values indicating greater cell viability.

### 2.5. Antibacterial Activity Evaluation

#### 2.5.1. Bacterial Susceptibility to AgNPs in Liquid Medium

The antimicrobial activity of the functionalized membranes was investigated against the following three biofilm-producing bacterial strains: *Pseudomonas aeruginosa* ATCC 27853 (PA), *Staphylococcus aureus* ATCC 29213 (SA), and a vancomycin-resistant clinical isolate of Enterococcus faecium (VRE), named Ef-5 [34].

The susceptibility of the strains to AgNPs dispersed in a liquid medium was investigated in advance to avoid problems due to growth inhibition induced by the nanoparticles released from the membranes in the subsequent tests. The minimum inhibitory concentration (MIC) of the AgNPs was evaluated by a microbroth dilution assay carried out in Cation-Adjusted Müller Hinton Broth (CAMHB) (Oxoid), according to the Clinical Laboratory Standards Institute guidelines [35]. AgNPs were tested at a maximum dose of 400 µg/mL, in accordance with previous studies [28]. Briefly, an initial inoculum of 5 × 10^5^ cells/mL was incubated at 37 ° with serial two-fold dilutions of AgNPs, and after 20 h, turbidity was visually assessed to evaluate bacterial growth.

#### 2.5.2. Biofilm Formation on Functionalized Membranes

The ability of the three strains (PA, SA, and VRE) to form biofilms on PCL membranes was then evaluated in comparison to the collagen and collagen + AgNP-functionalized membranes. Three batches of membranes (PCL, PCL + collagen, and PCL + collagen + AgNPs) with a diameter of 5 mm were prepared. Each membrane was incubated at 37° with orbital shaking (200 rpm) in 1 mL of culture medium (Luria–Bertani broth for *P. aeruginosa*, Brain Heart Infusion for the Gram-positive strains) with an initial inoculum of 107 cells/mL. After 24 h, the medium was removed, and each membrane was rinsed three times with 2 mL PBS to remove the remaining planktonic cells. The amount of biofilm was evaluated as described in [36].

The membranes were fixed in methanol for 20 min, dried, stained with 2% Hucker crystal violet (CV) for 15 min, and carefully rinsed with water to remove excess CV. Finally, the amount of CV solubilized from the dried membranes in 400 µL of 33% (*v/v*) CH3COOH was evaluated spectrophotometrically at 560 nm. Each test was performed in triplicate and repeated three times, always including the negative controls (membranes incubated in sterile medium).

### 2.6. Statistical Analysis

Statistical analyses were performed to evaluate the effects of different membrane formulations on biofilm inhibition and cell viability. For the biofilm inhibition assay, data from four independent replicates were combined and analyzed using a one-way analysis of variance (ANOVA) to identify significant differences among the tested membranes for each bacterial strain. Post hoc pairwise comparisons were conducted using Tukey’s Honest Significant Difference (HSD) test to determine which membrane pairs exhibited significant differences.

For the MTT assay, cell viability data were analyzed at the 24, 48, and 72-hour time points. One-way ANOVA was performed at each time point, followed by Tukey’s HSD test for pairwise comparisons when significant differences were identified. The significance level for all tests was set at *p* ≤ 0.05.

All statistical analyses were conducted using Python 3.11 with the SciPy and Stats models libraries. Data were checked for normality and homogeneity of variance to ensure the validity of the parametric tests.

## 3. Results and Discussion

The findings of this study present a comprehensive evaluation of electrospun nanofiber membranes composed of PCL, collagen, and AgNPs for potential use in advanced wound healing applications. The results indicate that the combination of structural support, biocompatibility, and antimicrobial functionality in these membranes could address some of the major challenges in wound care, including infection control and biofilm formation [7].

### 3.1. Membrane Characterization

#### 3.1.1. Membranes Morphology Characterization

**Figure 1:**
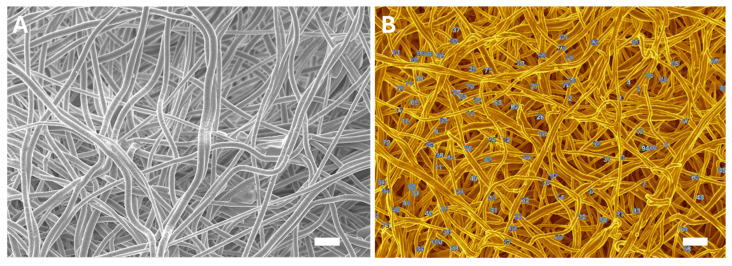
(**A**) SEM image of the PCL nanofibers; (**B**) measurement of nanofibers diameter. Scale bar is 3 µm.

The morphology of the electrospun nanofiber membranes was examined using SEM. The SEM images revealed that all membrane types, including PCL, collagen-coated PCL, collagen-citrate-coated PCL, and collagen-citrate-AgNP-coated PCL, exhibited a uniform, porous structure. The fibers were randomly oriented, mimicking the ECM, which is advantageous for promoting cell adhesion and proliferation [37]. The average fiber diameters ranged approximately between 350 and 550 nm (Figure 1 and Figure S1), aligning with the optimal size range reported in the literature [38] for promoting cellular activities such as adhesion and migration, which are crucial for wound healing. The slight variations in fiber diameter among the different membrane compositions were consistent with the optimized electrospinning parameters.

Confocal microscopy (Figure S2) demonstrated that the collagen coating was uniformly applied across the nanofiber membranes without compromising their structural integrity. This uniform coverage is critical for enhancing biocompatibility and improving cell–material interactions.

**Figure 2:**
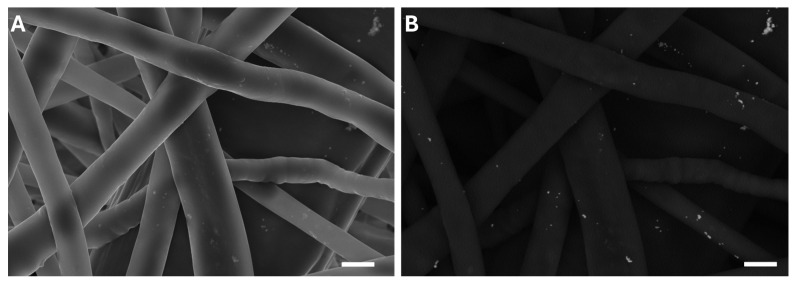
SEM images of silver nanoparticles on PCL nanofibers acquired with secondary electrons (**A**) and backscattered electrons (**B**). Scale bar is 1 µm.

SEM imaging in backscattered electron mode (Figure 2), however, revealed some heterogeneity in the distribution of AgNPs, with small clusters detected in certain areas. Although these clusters do not significantly impact the functionality of the membranes, achieving a more uniform coating remains a priority. Optimization efforts, such as centrifugation of the AgNP solution prior to coating, are currently underway to address this limitation.

**Figure 3:**
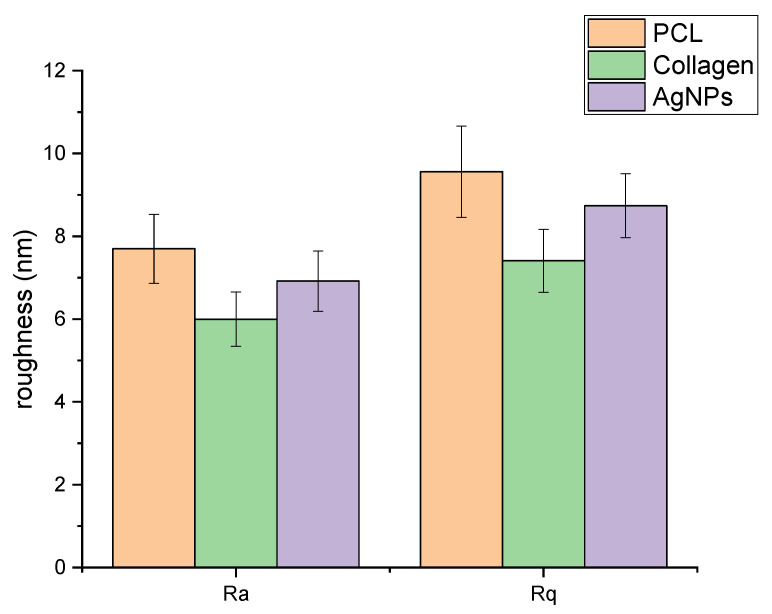
Data of nanofiber roughness expressed as Ra and Rq.

AFM further provided detailed information about the surface roughness of the membranes. AFM analysis showed that the collagen coating slightly decreased the surface roughness of the fibers; conversely, the coating with AgNPs increased surface roughness (Figure 3). This surface modification likely contributed to the observed increase in fibroblast adhesion and proliferation, supporting the hypothesis that surface topography plays a key role in cell–material interactions [39]. AFM also enabled the measurement of AgNP size, which was confirmed to be 50–100 nm, forming a bioactive surface that integrates antimicrobial properties with a structural framework supportive of cellular interactions. Additional SEM and AFM images (Figures S3 and S4) for all membrane formulations, including PCL, PCL + collagen, and PCL + collagen + AgNPs, illustrate the differences in surface morphology and roughness, offering a comprehensive understanding of the impact of each modification.

#### 3.1.2. Characterization of Membranes Wettability and Swelling

**Figure 4:**
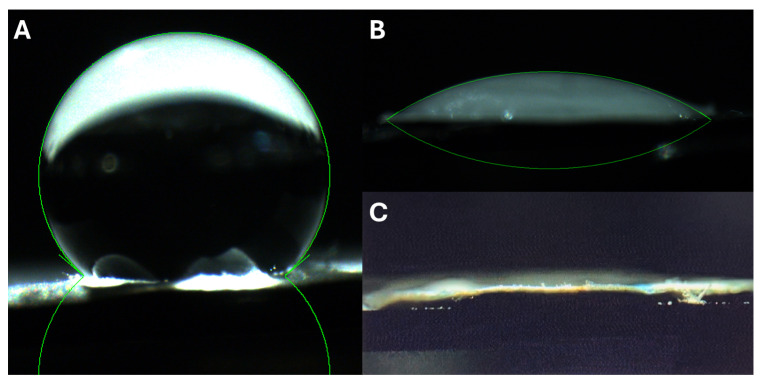
Water contact angle of PCL membranes (**A**), air plasma-treated PCL membranes (**B**), and collagen coated air plasma-treated membranes (**C**).

The surface hydrophilicity of the membranes was evaluated using contact angle measurements (Figure 4). The pure PCL membranes exhibited a relatively high contact angle (140° ± 2°), indicative of their hydrophobic nature. However, plasma treatment followed by collagen coating significantly reduced the contact angle, with plasma-treated membranes showing angles of 32° ± 6° and collagen-coated membranes achieving complete wettability (0°), confirming enhanced hydrophilicity. These modifications are crucial for wound healing, as a hydrophilic surface promotes cell adhesion and facilitates the absorption of wound exudate [40].

Water absorption tests further corroborated the membranes’ ability to maintain a moist wound environment, which is known to accelerate the healing process and reduce the risk of infection [41]. The swelling ratio of the air plasma-treated PCL membranes increased from 53% ± 4 for pure PCL to 140% ± 5, suggesting an improved fluid absorption capacity. Collagen coating further enhanced the swelling ratio to 164% ± 3, demonstrating the hydrophilic contribution of the collagen layer. These results highlight the role of surface modifications in optimizing the membrane’s functionality to effectively manage wound exudate, an essential property of wound dressings [42].

The final membrane formulation, PCL/Col/AgNPs, was also evaluated for its contact angle and swelling behavior. The addition of AgNPs to the PCL/Col membranes did not significantly affect either property, as the contact angle and swelling ratio remained consistent with those of the PCL/Col membranes. This outcome aligns with our expectations, as the small quantity of AgNPs primarily enhances antibacterial properties without substantially altering the surface chemistry or hydrophilicity governed by the collagen component.

Similar findings have been reported in the literature, where metallic nanoparticles at low concentrations exhibited negligible effects on these properties. For instance, Park et al. (2016) [43] and Yin et al. (2013) [44] demonstrated that the addition of AgNPs to composite membranes had a minimal impact on the contact angle and swelling behavior. The observed results validate the compatibility of AgNPs with the hydrophilic properties imparted by the collagen coating, ensuring the membranes’ functionality remains intact while providing additional antimicrobial benefits.

### 3.2. Membranes Biological Properties

#### Biocompatibility Testing

**Figure 5:**
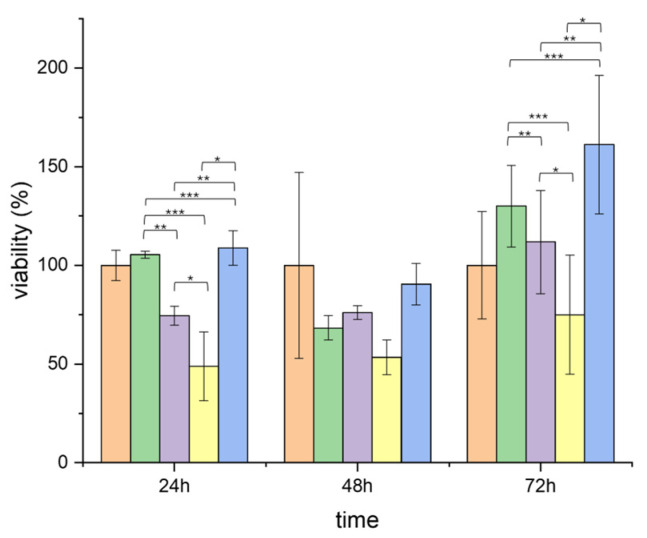
Cell viability expressed as a percentage relative to the positive control of cell growth (orange) for cells treated with PCL (green), PCL-col (violet), PCL-col-citrate (yellow), and PCL-col-citrate-AgNPs (blue) membranes. *: *p* < 0.05, **: *p* < 0.01, ***: *p* < 0.001.

The biocompatibility of the membranes was assessed using 3T3 murine fibroblasts, focusing on cell viability and proliferation over 24, 48, and 72 h. The MTT assay results (Figure 5) indicated that all membrane types supported cell growth, with absorbance values increasing over time for all formulations, reflecting active cell proliferation.

Collagen-coated membranes consistently exhibited the highest cell viability, suggesting that the collagen coating significantly enhanced the bioactivity of the fibers by providing additional binding sites for cell attachment. These findings align with previous research demonstrating that collagen, as a key component of the ECM, promotes cellular activities essential for wound healing by facilitating cell adhesion and proliferation [45,46].

The AgNP-functionalized membranes also displayed good biocompatibility, with minimal reduction in cell viability compared to the PCL/Col membranes. This result highlights the careful optimization of AgNP concentrations, which were sufficient to provide antimicrobial effects without inducing significant cytotoxicity. The ability of the membranes to balance antibacterial efficacy with cellular safety is particularly noteworthy, as AgNPs, while effective against a broad range of pathogens, are known to generate ROS at high concentrations, potentially leading to cytotoxic effects [47]. By incorporating AgNPs at controlled concentrations, the membranes in this study achieve a balance between antimicrobial efficacy and cellular compatibility. This balance is critical for applications in wound healing, where maintaining fibroblast viability is essential for tissue regeneration. The promising biocompatibility profiles observed for AgNP-containing membranes, particularly during the early stages of exposure, suggest their suitability for biomedical applications. Further optimization of the membrane composition may enhance both efficacy and safety, paving the way for advanced wound dressing solutions.

### 3.3. Membranes Antibacterial Activity

#### 3.3.1. Broth Dilution Test

The antibacterial activity of the AgNPs was evaluated using a microbroth dilution assay to determine the MIC for the following three bacterial strains: *Pseudomonas aeruginosa* (PA), *Vancomycin*-resistant *Enterococcus* (VRE), and *Staphylococcus aureus* (SA). The results (Table 1) revealed that PA was the most susceptible, with complete inhibition observed at 50 µg/mL. VRE also demonstrated sensitivity to AgNPs at 100 µg/mL, while SA exhibited resistance, even at higher AgNP concentrations (>400 µg/mL).

These findings indicate that the antibacterial activity of the AgNPs varies depending on the bacterial strain, which is consistent with differences in their cell wall structures and resistance mechanisms. The AgNPs exhibited the highest activity against PA and VRE, two pathogens known for their antibiotic resistance and biofilm-forming abilities. The effectiveness of AgNPs against these strains is significant, as PA and VRE are notorious for their ability to evade both antimicrobial agents and the host’s immune response [48].

The antibacterial effects of AgNPs are likely mediated through multiple mechanisms, including the disruption of bacterial cell membranes, generation of ROS, and interference with DNA replication. These mechanisms, which target fundamental bacterial processes, make AgNPs a potent component for wound dressings [49]. However, the limited efficacy observed against SA, with an MIC exceeding 400 µg/mL, highlights the need for the further refinement of the current formulation. SA’s resistance may be attributed to its biofilm-forming capabilities, efflux pumps, or modifications of cellular targets, which are known mechanisms in bacterial resistance to silver nanoparticles [50].

To address this limitation, future research could focus on enhancing the antimicrobial profile of the membranes by incorporating additional agents, such as natural plant extracts or antibiotics. Such combinations may be synergized with AgNPs to overcome resistance mechanisms and provide broader-spectrum antimicrobial activity. Despite the reduced efficacy against SA, the demonstrated effectiveness against PA and VRE underscores the potential of AgNP-functionalized membranes for managing wounds infected with resistant pathogens.

#### 3.3.2. Biofilm Inhibition

**Figure 6:**
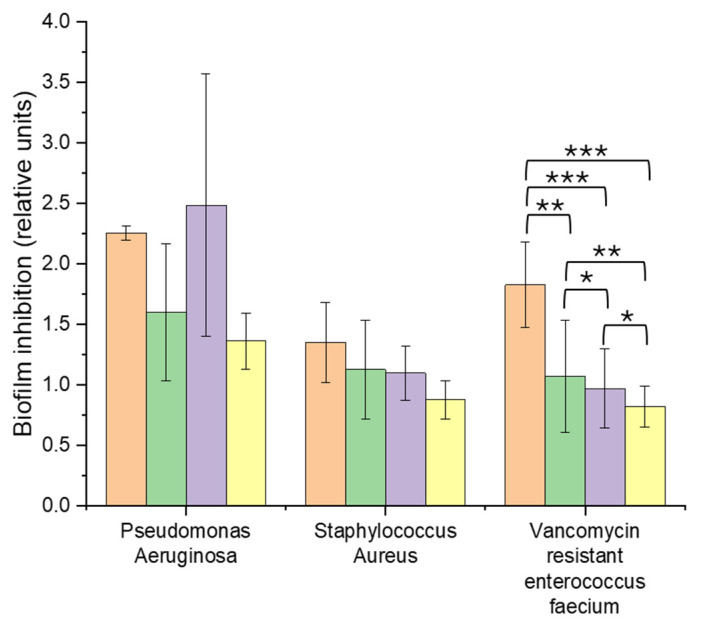
Inhibition of biofilm growth, derived from absorbance measurements in the presence of PCL (orange), PCL-col (green), PCL-col-citrate (violet), and PCL-col-citrate-AgNPs (yellow) membranes. *: *p* < 0.05, **: *p* < 0.01, ***: *p* < 0.001.

The ability of the membranes to inhibit biofilm formation was a critical aspect of the antibacterial evaluation. The results (Figure 6) showed that the AgNP-functionalized membranes significantly reduced biofilm formation in PA and VRE by 39.74% and 55.10%, respectively. Compared to non-functionalized membranes, bacterial biofilm inhibition was approximately 40–50%, underscoring the effectiveness of AgNPs in preventing biofilm development. However, the membranes exhibited limited biofilm inhibition against *Staphylococcus aureus* (SA), with a reduction of 30.75%.

The biofilm inhibition capabilities of the AgNP-functionalized membranes are particularly noteworthy, given the challenges posed by biofilms in wound management. Biofilms are highly resistant to conventional treatments and play a significant role in chronic wound infections [6]. The observed efficacy against PA and VRE aligns with previous studies, which suggest that AgNPs can disrupt quorum sensing, the bacterial communication system that regulates biofilm formation [51]. By interfering with this process, AgNPs prevent the establishment and maturation of biofilms, thereby reducing the risk of chronic infections and improving wound healing outcomes.

The ability of the membranes to inhibit biofilm formation has critical clinical implications. Effective biofilm prevention could reduce the need for systemic antibiotics, an important consideration in the context of rising antibiotic resistance. Furthermore, the enhanced biofilm inhibition observed against VRE highlights the potential of AgNP-based membranes for addressing infections caused by resistant pathogens. However, the limited inhibition observed for SA underscores the importance of strain-specific optimization. Variability in biofilm inhibition across strains may be attributed to differences in bacterial cell wall structure, quorum sensing pathways, and resistance mechanisms.

Overall, these findings demonstrate the potential of AgNP-functionalized membranes as a targeted approach to combat biofilm formation in resistant pathogens. While the results are promising, future studies should explore strategies to enhance biofilm inhibition for strains like SA, such as the incorporation of complementary antimicrobial agents or the further optimization of AgNP concentrations. This study provides valuable insights for the development of advanced wound dressings capable of mitigating biofilm-associated complications in chronic wound infections.

### 3.4. Analysis of the Data

#### 3.4.1. Analysis of Cell Viability (MTT Assay)

The effect of various membranes on cell viability was assessed using the MTT assay over 24, 48, and 72 h. A one-way ANOVA was conducted. The results indicated statistically significant differences among membranes at 24 h (F = 24.68, *p* = 0.0002) and 72 h (F = 5.64, *p* = 0.022), whereas no significant differences were observed at 48 h (F = 1.23, *p* = 0.36).

A Tukey HSD post hoc analysis revealed specific membrane combinations that contributed to the observed differences. At 24 h, membranes containing AgNPs demonstrated reduced cytotoxicity compared to others. At 72 h, the differences between membranes narrowed, although certain formulations maintained superior performance in terms of cell viability.

Interestingly, membranes containing AgNPs exhibited reduced cytotoxicity at 24 h, even compared to positive controls. This effect may be attributed to the ability of AgNPs to stimulate cellular metabolism, possibly through mild oxidative stress that activates mitochondrial activity and adaptive cellular responses. Some studies suggest that AgNPs may influence ECM, cytokines, and growth factors [52,53,54].

Additionally, the antibacterial properties of AgNPs may reduce microbial contamination, creating a more favorable environment for fibroblast growth. Further studies are needed to explore these mechanisms, including investigations into silver ion release, ROS generation, and mitochondrial function.

#### 3.4.2. Biofilm Inhibition Analysis

The effects of various membrane formulations on biofilm inhibition were evaluated for the following three bacterial strains: PA, SA, and VRE. A one-way ANOVA was conducted using raw data from four independent tests. The analysis revealed significant differences among the membranes for VRE (F = 6.78, *p* = 0.006), while no significant differences were observed for PA (F = 2.17, *p* = 0.17) and SA (F = 1.70, *p* = 0.22).

The post hoc Tukey HSD analysis for VRE highlighted the superior biofilm inhibition efficacy of membranes containing AgNPs compared to other formulations (*p* ≤ 0.05).

#### 3.4.3. Clinical Implications and Future Directions

These findings have important clinical implications. The balance between biocompatibility and antimicrobial efficacy supports the potential use of AgNP-functionalized membranes in advanced wound care, including chronic wound management and post-surgical wound care. The significant biofilm inhibition observed in PA and VRE suggests that these membranes could play a key role in preventing infections in wounds susceptible to biofilm formation. However, the limited effectiveness against SA indicates that further optimization is needed, possibly through the incorporation of additional antimicrobial agents or the development of controlled-release systems [55].

#### 3.4.4. Limitations

While the current study relied on indirect methods (SEM images in backscattered electron mode) to confirm the characteristics of AgNPs, the use of a standardized synthesis method and literature validation provides confidence in the nanoparticles’ quality. Future studies will incorporate direct TEM imaging to complement these findings and further validate the morphological properties of the AgNPs.

While the study demonstrates promising results, the variability in certain experimental data (Figure 5 and Figure 6) underscores the need for further investigations to refine and validate these findings. Ongoing experiments are focused on addressing these margins of error to ensure greater consistency and reliability in future studies.

## 4. Conclusions

This research advances plastic surgery and wound healing by introducing a novel multifunctional nanofiber membrane that combines biocompatibility and antimicrobial protection. The electrospun nanofibers closely mimic the ECM, fostering an ideal environment for cell attachment and proliferation, which is crucial for tissue regeneration. The integration of AgNPs into the membrane enhances antimicrobial efficacy, potentially reducing the reliance on systemic antibiotics and addressing antibiotic resistance, an especially critical factor in chronic wounds where biofilm formation is a significant hurdle. While these promising findings highlight the membranes’ biocompatibility and biofilm-inhibiting properties, the variability in antimicrobial performance signals a need for ongoing research to refine these materials for clinical application. Future work should involve in vivo testing to assess real-world effectiveness and investigate additional antimicrobial agents to broaden the membranes’ activity spectrum, ensuring their suitability for diverse wound care applications.

## Figures and Tables

**Table 1:** AgNP broth dilution assay.

SA	0 µg/mL	6.25 µg/mL	12.5 µg/mL	25 µg/mL	50 µg/mL	100 µg/mL	200 µg/mL	400 µg/mL
set 1	+	+	+	+	+	+	+	+
set 2	+	+	+	+	+	+	+	+
set 3	+	+	+	+	+	+	+	+
set 4	+	+	+	+	+	+	+	+
set 5	+	+	+	+	+	+	+	+
VRE	0 µg/mL	6.25 µg/mL	12.5 µg/mL	25 µg/mL	50 µg/mL	100 µg/mL	200 µg/mL	400 µg/mL
set 1	+	+	+	+	+	−	−	−
set 2	+	+	+	+	+	−	−	−
set 3	+	+	+	+	+	−	−	−
set 4	+	+	+	+	+	−	−	−
set 5	+	+	+	+	+	−	−	−
PA	0 µg/mL	6.25 µg/mL	12.5 µg/mL	25 µg/mL	50 µg/mL	100 µg/mL	200 µg/mL	400 µg/mL
set 1	+	+	+	+	−	−	−	−
set 2	+	+	+	+	−	−	−	−
set 3	+	+	+	+	−	−	−	−
set 4	+	+	+	+	−	−	−	−
set 5	+	+	+	+	−	−	−	−

Positive (+): turbidity indicating growth; negative (−): no turbidity indicating absence of growth. SA: *Staphylococcus aureus*; PA: *Pseudomonas aeruginosa*; VRE: vancomycin-resistant *Enterococcus*.

## Data Availability

The raw data supporting the conclusions of this article will be made available by the authors upon request.

## Supplementary Materials

The following supporting information can be downloaded at: https://www.mdpi.com/article/10.3390/membranes15020039/s1, Figure S1: nanofibers average diameter; Figure S2: nanofibers fluorescence imaging; Figure S3: nanofibers surface roughness SEM imaging; Figure S4: nanofibers surface roughness AFM imaging.
