# Supplementary material for: Electrospun Collagen-Coated Nanofiber Membranes Functionalized with Silver Nanoparticles for Advanced Wound Healing Applications
Source: Membranes (Basel). 2025 Feb 1;15(2):39. doi: 10.3390/membranes15020039 (PMC11857158; doi:10.3390/membranes15020039)
Supplement: Supplementary file 1 [file membranes-15-00039-s001.zip › membranes-3391183-supplementary.pdf]

Electrospun Collagen-Coated Nanofiber Membranes Functionalized with Silver Nanoparticles for Advanced Wound Healing Applications

Martin Iurilli, Davide Porrelli, Gianluca Turco, Cristina Lagatolla, Alvise Camurri Piloni, Barbara Medagli, Vanessa Nicolin and Giovanni Papa

SUPPLEMENTARY MATERIALS

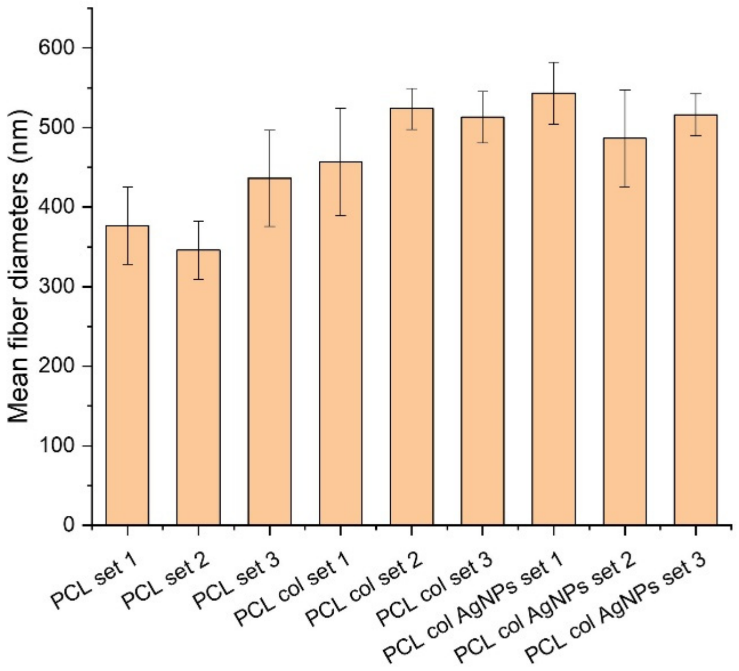

Figure S1. Average diameters of nanofibers based on SEM images.
PCL: Polycaprolactone; PCL col: Polycaprolactone with collagen; PCL col AgNPs: Polycaprolactone with collagen and silver nanoparticles

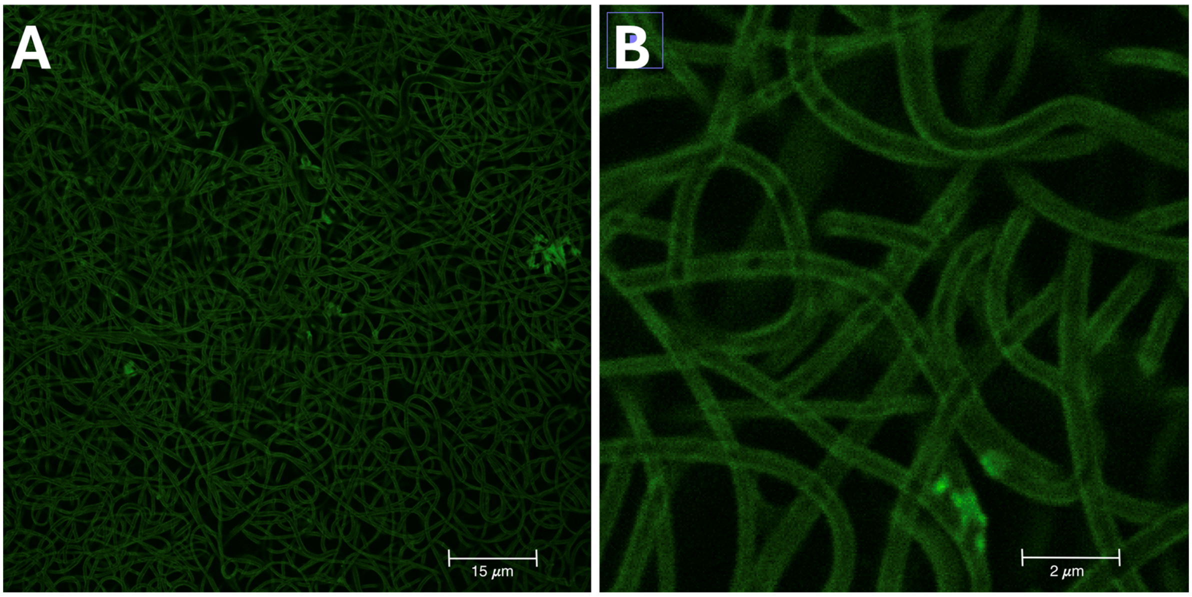

Figure S2. Confocal microscopy images using bovine Type I collagen labeled with fluorescein isothiocyanate (FITC). These images confirm a uniform distribution of collagen across the nanofiber membranes without altering the fiber architecture.

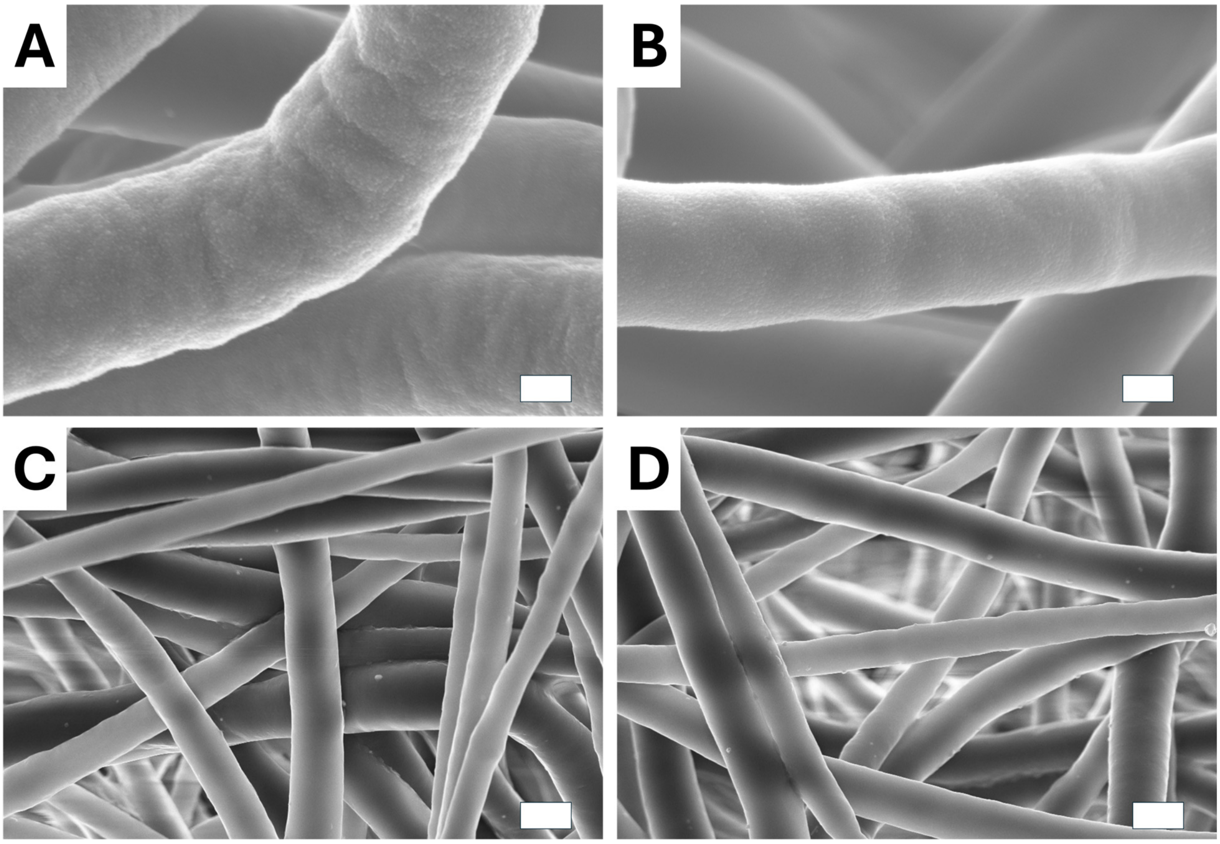

Figure S3. SEM images illustrating the detail of the nanofiber surface morphology and roughness. A: Polycaprolactone; B: Polycaprolactone with collagen; C & D: Polycaprolactone with collagen and silver nanoparticles. Scale bar is 200 nm for A and B, and 1 μm for C and D

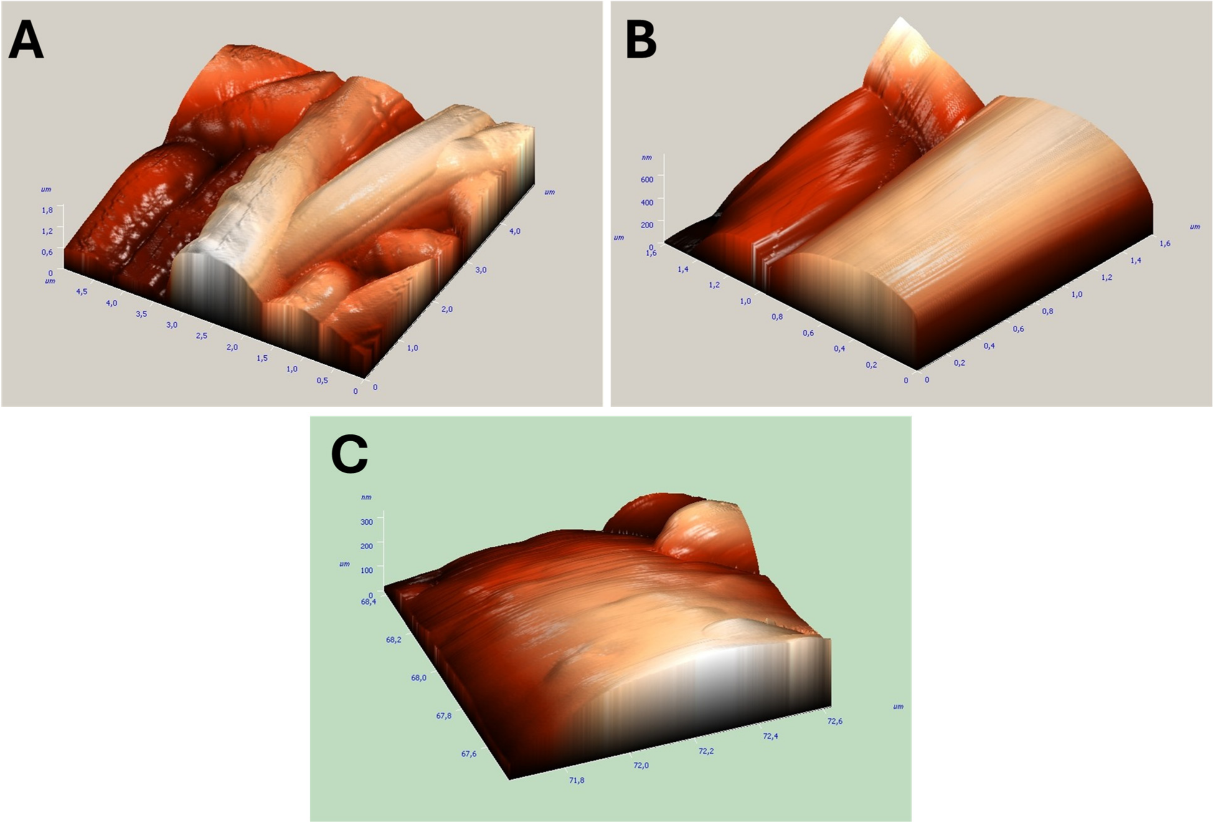

Figure S4. AFM images illustrating the detail of the nanofiber surface morphology and roughness. A: Polycaprolactone; B: Polycaprolactone with collagen; C: Polycaprolactone with collagen and silver nanoparticles.
